# Genomic Analysis of Consecutive *Acinetobacter baumannii* Strains From a Single Patient

**DOI:** 10.3389/fmicb.2018.02840

**Published:** 2018-11-27

**Authors:** Sun Ju Kim, Yae-Jean Kim, Kwan Soo Ko

**Affiliations:** ^1^Department of Molecular Cell Biology, Samsung Medical Center, Sungkyunkwan University School of Medicine, Suwon, South Korea; ^2^Department of Pediatrics, Samsung Medical Center, Sungkyunkwan University School of Medicine, Seoul, South Korea

**Keywords:** *Acinetobacter baumannii*, whole genome sequencing, haemophagocytic lymphohistiocytosis, within-host evolution, re-infection

## Abstract

*Acinetobacter baumannii* is one of the most important nosocomial pathogens, and thus it is required to investigate how it disseminate in hospitals and infect patients. We performed whole genome sequencing for 24 *A. baumannii* strains isolated successively from the blood of a single patient to evaluate whether repeated infections were due to re-infection or relapse infection and to investigate within-host evolution. The whole genome of the first strain, BL1, was sequenced *de novo* using the PacBio RSII system. BL2–BL24, were sequenced with an Illumina Hiseq4000 and mapped to the genome sequences of BL1. We identified 42 single-nucleotide variations among the strains. The SNVs differentiated the strains into three groups, BL1, BL2–BL16, and BL17–BL24, indicating that the patient suffered from re-infections or co-infections by similar, but different strains. The results also showed that *A. baumannii* strains in each group were rather stable at the genomic level. Our study emphasizes the importance of intensive infection control.

## Introduction

*Acinetobacter baumannii* is an important opportunistic pathogen associated with respiratory infections, bacteraemia, meningitis, and wound infection. The increasing antibiotic resistance of *A. baumannii* due to dissemination of multidrug-resistant (MDR) strains has posed a great threat to hospital patients (Munoz-Price and Weinstein, 2008). It is difficult to treat MDR *A. baumannii* infections because of relapse infection or re-infection.

We recently reported successively isolated MDR *A. baumannii* from a single patient who died from haemophagocytic lymphohistiocytosis (Choi et al., 2017). In that study, *A. baumannii* isolates from blood were divided into two groups based on pulsed-field gel electrophoresis, suggesting that the strains isolated in the latter period did not recur, but that patients may be re-infected with a different strain isolated in the former period. However, we could not confirm the re-infection because of the limitations of the methods and we could not investigate genomic changes during infection.

Whole genome sequencing (WGS) enables exploration of the dynamics and genomic evolution of bacterial pathogens during infection (Didelot et al., 2016). For example, WGS was applied to track the within-host evolution of *Staphylococcus aureus, Helicobacter pylori, Pseudomonas aeruginosa*, and *Mycobacterium tuberculosis* (Kennemann et al., 2011; Young et al., 2012; Marvig et al., 2014; Witney et al., 2017). For *A. baumannii*, WGS was performed a few times in a longitudinal study of the colistin resistance mechanism (Snitkin et al., 2013). In this study, we performed WGS for successive *A. baumannii* strains from the patient to differentiate re-infection from relapse infection at the genome level and investigate the within-host evolution of *A. baumannii*.

## Materials and Methods

Of the 38 isolates collected from various specimens in a single patient deceased from haemophagocytic lymphohistiocytosis (Choi et al., 2017), 24 isolates (BL1–BL24) from the blood were included in this study. As shown in our previous study, the first blood isolate BL1 was collected on April 12, 2012, BL2–BL16 were isolated between May 17 and June 18, 2012, and BL17–BL24 were isolated from August 16–31, 2012.

Total DNA of the strains was extracted by using G-spin^TM^ Genomic DNA Extraction Kit for Bacteria (iNtRON Biotechnology, Inc., South Korea). Library sample of BL1 were prepared as 20 kb SMRTbell^TM^ Templates (Pacific Biosciences, Menlo Park, CA, United States) and samples of BL2-24 were constructed by using TruSeq DNA PCR-Free (Illumina, Inc., San Diego, CA, United States).

BL1 was sequenced to prepare a draft genome for use as a reference of comparison using the PacBio RSII system (Pacific Biosciences, Menlo Park, CA, United States). HGAP3 and Quiver were used for *de novo* assembly and confirmation. The annotated whole genome sequence of BL1 has deposited in the GenBank BioSample, under the accession number CP025266 (chromosome) and CP025267 (plasmid). Open reading frames were predicted and annotated using the Prokka (v1.12b, Seemann, 2014). Prophages both in the chromosome and plasmid were detected with PHASTER (Arndt et al., 2017). Antibiotic resistance genes were searched and confirmed by the RAST server (Aziz et al., 2008) and Resistance gene identifier (Jia et al., 2017). BL2–BL24 were sequenced using the Illumina HiSeq4000 (Illumina, Inc., San Diego, CA, United States) and were mapped to the genome sequences of BL1 to trace genomic changes from BL1. The numbers of total reads, coverage, and depth of short-read sequencing using the Illumina were 18,008,884 to 28,047,056, 99.68 to 100%, and 407 to 627, respectively. All strains have each paired-ends larger than three gigabytes with N50 from five to eight mega base pairs. Snippy was applied to detect single-nucleotide variations (SNVs) by threshold of 0.7. (Seemann, 2014). SNVs among the genes characterizing those three periods: *acdP*, CV094_04170 encoding hypothetical protein, *mvaB, ampC, ureC* were confirmed by Sanger sequencing. All SNVs on those genes were confirmed except frameshift of *ureC* in BL3, which showed relatively low evidence hit per depth. To predict whether the identified amino acid substitutions were likely to affect protein function, we used the PROVEAN web server (Choi and Chan, 2015). A phylogenetic tree was drawn based on 30 SNVs by the neighbor-joining method (MEGA 5.10) with an outgroup as *A. baumannii* ST138 strain A1296 (GenBank accession number, CP018332.1).

The genome sequences of BL1–BL24 generated for this study can be found in the GenBank.^1^

## Results

The chromosome of *A. baumannii* BL1 comprises 3,983,848 base pairs (bp), which encodes 3,852 coding sequence, 18 tRNA, 1 tmRNA, and 73 rRNA genes. Forty-five insertion sequences (ISs) were identified, 11 of which were intact. Four predicted prophages were also found. Three intact prophages (47.6–52.6 kb in length) were similar to *Acinetobacter* phage YMC/09/02/B1251_ABA_BP, and an incomplete prophage (28.9 kb in length) showed similarity to *Enterobacteria* phage VT2phi_272 (Supplementary Table 1). The overall G+C content was 39.1%. Based on the results of a BLAST search, *A. baumannii* KBN10P02143 (GenBank accession number CP013924) (Lee Y.W. et al., 2016) from South Korea showed the most similar genome to the BL1 strain. Twenty-seven antibiotic resistance genes, including *bla*_OXA-66_ and *bla*_OXA-23_, were identified in the chromosome (Supplementary Table 1). In our previous study, we reported that the successively isolated *A. baumannii* strains belonged to ST138 according to multilocus sequence typing analysis (Choi et al., 2017). *In silico* analysis in this study revealed that the strains belonged to ST191 based on the difference in a nucleotide from ST138 at the *gpi* locus. The misidentification may have resulted from obscurity in sequencing near the primers, which has been reported previously (Hamidian et al., 2017).

One plasmid was also sequenced. The plasmid pBL1 was found to be 74,241 bp in size with an average G+C content of 39.0%. It contains 100 predicted ORFs and one IS. The plasmid was highly similar to that of *A. baumannii* strain 15A5, p15A5_1 from South Korea (GenBank accession number CP020573.1), differing in only one nucleotide site.

**FIGURE 1:**
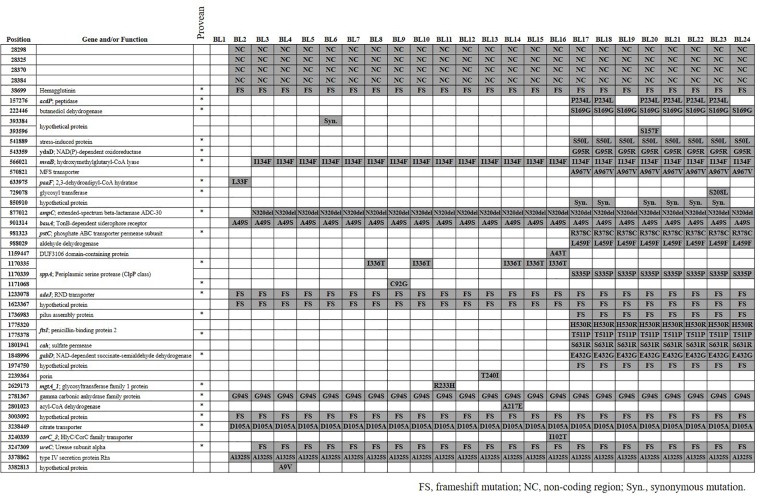
SNVs among 24 *Acinetobacter baumannii* strains. SNV positions were based on the genome sequences of BL1 and genes were annotated using the Prokka. In the PROVEAN column, an asterisk (^∗^) indicates that the amino acid alteration was predicted to affect protein function. FS, frameshift mutation; NC, nucleotide variation in non-coding region; Syn, synonymous mutation.

We identified a total of 42 SNVs among the 23 *A. baumannii* strains from blood (BL2–BL24) compared with the genome sequences of BL1 (Figure 1). While 38 SNVs were identified in 34 coding genes including 6 hypothetical genes, four were found in non-coding regions. Multiple SNVs were identified only in *sppA* encoding putative signal peptide peptidase, *ftsI* encoding peptidoglycan synthase, and a hypothetical protein. Among the 38 SNVs found in coding genes, 36 were non-synonymous mutations and two were synonymous. Seven non-synonymous mutations resulted in frame shifts. The plasmid and phages that were identified in BL1 were present in all the other strains, BL2–BL24. We took the discarded unmapped reads and tried to assemble them *de novo* using using SPAdes (Bankevich et al., 2012) with *k-mer* size 31. Similarity of the contigs over 100 bp were verified through nr/nt database of NCBI, and all 23 strains (BL2–BL24) had a highly similar contig. The contig was 8753 bp-long as a median, and 99% matched with pA85-2 of *A. baumannii* strain A85 (CP021786.1).

Twelve SNVs including four in non-coding regions occurred commonly in the BL2–BL24 strains, separating them from the BL1 strain. Three SNVs, such as I134F (position 566021) in *mvaB* encoding hydroxymethyl glutaryl-CoA lyase, were identified in the BL3–BL24 strains. Twelve SNVs were commonly identified in strains BL17–BL24. P234L (position 157276) in *acdP* and a synonymous mutation (position 850910) in a hypothetical protein were also found fromBL17, but not in BL19 and BL24. Eleven SNVs were found in only one strain, such as in S208L (position 729078) in a gene encoding glycosyl transferase.

**FIGURE 2:**
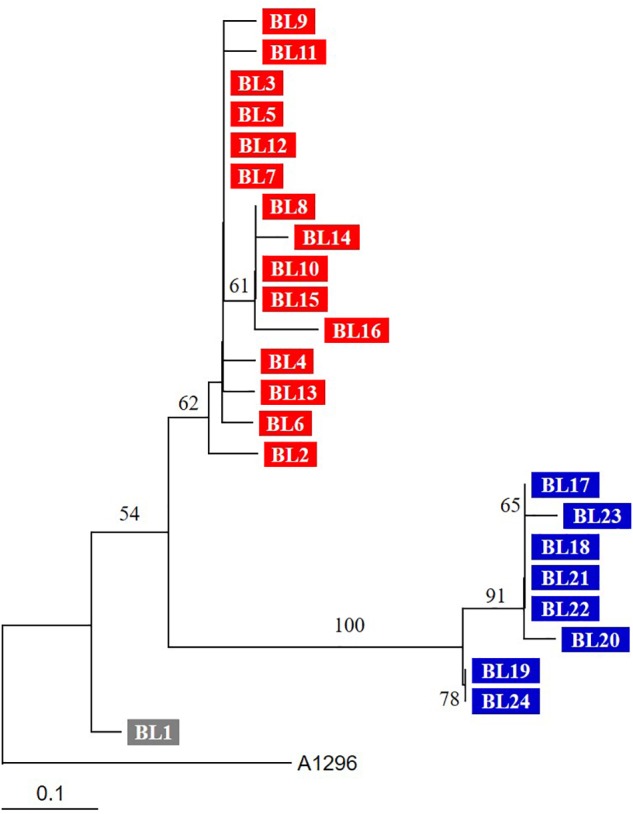
Phylogenetic tree based on SNVs of genome sequences. The tree was constructed by the neighbor-joining method. Three groups (BL1, BL2–BL16, and BL17–BL24) are indicated by different colors. The tree was reconstructed by the neighbor-joining method (MEGA 5.10), and *A. baumannii* ST138 strain A1296 was used as an outgroup. Numbers on branching nodes are percentages of 1,000 bootstrap replications; only values ≥ 50% are shown. The scale bar represents one substitution per 10 SNVs.

Based on PROVEAN, 23 SNVs were predicted to affect the function of proteins (Figure 1). The phylogenetic tree based on SNVs showed that they are grouped in two different clusters and the number of SNVs of each (Figure 2). BL1, the first isolated and reference strain, was also differentiated from BL2–BL16.

## Discussion

A patient who died from haemophagocytic lymphohistiocytosis might have been re-infected by another *A. baumannii* strain (Choi et al., 2017). Sixteen SNVs, including 15 non-synonymous substitutions, were identified only in strains BL17–BL24. The *A. baumannii* strains from the second period (BL17–BL24) were genetically different from those in the first period, although they shared the same genotype as demonstrated by multilocus sequence typing (ST191). In addition, BL1, the strain first isolated from the blood, may have been different from the others. Thus, the patient may have suffered from repeated infection by similar but different strains. The re-infected strains may have originated from the hospital environment or other patients. *A. baumannii* ST191 belonging to global clone 2 (GC2) is one the most prevalent clone in South Korea (Selasi et al., 2015; Kim et al., 2017). WGS analysis indicated that multiple sub-clones exist in the same intensive care unit and may invoke re-infection (Wright et al., 2014; Schultz et al., 2016). Our results emphasize the need for more intensive infection control to prevent re-infections in intensive care units.

However, another possibility was also proposed: multiple strains co-infected the patient. That is, two major groups of strains (BL2–BL16 and BL17–BL24) were infecting the patient at the same time, but only a single strain might be used for sequencing and another strain occurring at a low frequency are unlikely to have been sequenced. This is supported by the PFGE data that the PFGE pattern of BL2–BL16 was present in strains from other isolation source (wound, tracheal aspirate, and skin swab) throughout the study period. However, it is certain that main strain causing bloodstream infection has changed in the third period.

Although we previously showed that the strains in the second period had lower survival rates following exposure to human serum (Choi et al., 2017), our results in this study suggest that further physiological or biochemical traits exist because many genes with SNVs found only in the strains of the second period encode proteins with important functions. Based on PROVEAN prediction, the functions of many proteins were affected: Zn-dependent dipeptidase, butanediol dehydrogenase, stress-induced protein, phosphate transport system permease protein PstC, penicillin-binding protein 2 FtsI, and succinate-semialdehyde dehydrogenase. Further studies on the mechanisms by which the variations in these genes affect the physiological and biochemical features of *A. baumannii* would provide insight into the virulence and adaptive traits within hosts. In addition, we observed the predominance of non-synonymous mutation among SNVs in this study. In a recent study, much higher synonymous substitutions in recently emerged clones were reported (Castillo-Ramírez et al., 2011). In the recently emerged clones, genetic recombination may occur frequently and the efficiency of selection reduced. Based on the findings, our results suggested that *A. baumannii* strains in our study may not be under high genetic recombination and selection of clone.

In our study, BL17 and BL20–BL23 were susceptible to amikacin (Choi et al., 2017). While all strains except BL17 and BL20–BL23 have Tn*6180* bearing *armA*, which encodes 16S rRNA methylase and is associated with aminoglycoside resistance, Tn*6180* was not found in the BL17 and BL20–BL23. Thus, BL17 and BL20–BL23 become susceptible to amikacin by losing Tn*6180* bearing *armA*.

Our results also showed that *A. baumannii* strains were rather stable within the host at the genomic level. This finding is contrary to the results of previous studies (Liu et al., 2014; Graña-Miraglia et al., 2017), in which high genetic variations were identified in *A. baumannii* strains. Thirteen SNVs were identified in strains BL2–BL16, and only four SNVs existed among the strains from the second period (BL17–BL24). We also identified some sporadic variations in several strains. Genetic reversion may occur under specific circumstances (Lee J.Y. et al., 2016) but is rare. Thus, the sporadic SNVs may have been generated by selection bias. A clinician or researcher may isolate one strain from a few colonies and store it, although bacterial variants of the same genetic background may exist in the host. This is supported by the diverse-community model, in which several strains with polymorphisms coexist and compete within individuals (Lieberman et al., 2016). Thus, the strain isolated successively from a single patient may show genetic variations from other strains. Otherwise, the variations may be deleterious, and thus the strains with variations would be removed from the bacterial populations. However, only three of the 11 SNVs may be deleterious, as predicted by PROVEAN; C92G (position 1171068) in *sppA*, R233H (position 2629173) in *mgtA_1* encoding glycosyl transferase family 1 protein, and A217E (position 2801023) in a gene encoding acyl-CoA dehydrogenase.

Our approach using short-read sequencing method have some limitations. In the short-read sequencing, any re-arrangements and gene duplications may be missed. Thus, we analyzed further to overcome the limitations: confirmation of SNVs by Sanger sequencing, unmapped region on reference and vanishing of amikacin resistance among strains in the third period, and *de novo* assembly of unmapped reads.

In summary, we confirmed that the patient, deceased because of haemophagocytic lymphohistocytosis, had been re-infected at least three times or co-infected by *A. baumannii* strains. Despite genomic stability within the host, several SNVs among the strains were predicted to affect protein function.

## Author Contributions

SJK performed the experiments, analyzed the data, and wrote the manuscript. Y-JK provided the experimental materials, analyzed the data, and wrote the manuscript. KSK designed the experiments, analyzed the data, and wrote the manuscript.

## Conflict of Interest Statement

The authors declare that the research was conducted in the absence of any commercial or financial relationships that could be construed as a potential conflict of interest.

## Footnotes

1 https://www.ncbi.nlm.nih.gov/bioproject/PRJNA419431
