# Supplementary material for: Genomic Analysis of Consecutive Acinetobacter baumannii Strains From a Single Patient
Source: Front Microbiol. 2018 Nov 27;9:2840. doi: 10.3389/fmicb.2018.02840 (PMC6277775; doi:10.3389/fmicb.2018.02840)
Supplement: Supplementary file 1 [file Table_1.docx]

**Supplementary Table 1**. Resistance genes identified in chromosome of *A. baumannii* BL1

| **Gene** | **Start** | **Stop** | Resisistance |
| --- | --- | --- | --- |
| *adeS* | 120044 | 121117 | Multidrug efflux pump |
| *adeR* | 121149 | 121892 | Multidrug efflux pump |
| *adeA* | 122038 | 123228 | Multidrug efflux pump |
| *mexF* | 123225 | 126335 | Multidrug efflux pump |
| *bla*_OXA-66_ | 403655 | 404479 | Carbapenems |
| *msrE* | 681873 | 683348 | Macrolides |
| *armA* | 685647 | 686420 | Aminoglycosides |
| *sul1* | 689765 | 690604 | Sulphonamides |
| *aadA23* | 691109 | 691888 | Aminoglycosides |
| *catB8* | 691958 | 692590 | Chloramphenicol |
| *aac(6')-Ib7* | 692683 | 693315 | Aminoglycosides |
| *abeS* | 796382 | 796711 | Multidrug efflux pump |
| *adeF* | 801249 | 802469 | Multidrug efflux pump |
| *mdsB* | 802476 | 805655 | Multidrug efflux pump |
| *adeH* | 805668 | 807116 | Multidrug efflux pump |
| *bla*_ADC-30_ | 876056 | 877207 | Cephalosporins |
| *adeI* | 1339887 | 1341137 | Multidrug efflux pump |
| *mexF* | 1341150 | 1344326 | Multidrug efflux pump |
| *adeK* | 1344326 | 1345780 | Multidrug efflux pump |
| *abeM* | 2515358 | 2516704 | Multidrug efflux pump |
| *bla*_OXA-23_ | 2823734 | 2824555 | Carbapenems |
| *mexT* | 3240766 | 3241755 | Multidrug efflux pump |
| *bla*_TEM-1_ | 3399451 | 3400311 | Beta-lactams |
| *aac(1)* | 3407823 | 3408356 | Aminoglycosides |
| *aadA23* | 3409950 | 3410729 | Aminoglycosides |
| *sul1* | 3411234 | 3412073 | Sulphonamides |
| *adeN* | 3817203 | 3817856 | Multidrug efflux pump |
